# Functional evaluations comparing the double-tract method and the jejunal interposition method following laparoscopic proximal gastrectomy for gastric cancer: an investigation including laparoscopic total gastrectomy

**DOI:** 10.1007/s00595-018-1699-7

**Published:** 2018-08-29

**Authors:** Eiji Nomura, Hajime Kayano, Sang-Woong Lee, Masaru Kawai, Takashi Machida, Soichiro Yamamoto, Kazuhito Nabeshima, Kenji Nakamura, Masaya Mukai, Kazuhisa Uchiyama

**Affiliations:** 1 Department of Gastroenterological and General Surgery, Tokai University Hachioji Hospital, 1838 Ishikawa-machi, Hachioji, Tokyo, 192-0032 Japan; 2 Department of General and Gastroenterological Surgery, Osaka Medical College, 2-7 Daigaku-machi, Takatsuki, Osaka 569-8686 Japan; 3 Department of Gastroenterological and General Surgery, Tokai University School of Medicine, 143 Shimokasuya, Isehara, Kanagawa 259-1193 Japan

**Keywords:** Gastric cancer, Laparoscopic proximal gastrectomy, Double tract reconstruction, Jejunal interposition reconstruction, Quality of life

## Abstract

**Purpose:**

Functional outcomes were prospectively compared between two types of reconstruction [double tract (L-DT; *n* = 15) and jejunal interposition (L-JIP; *n* = 15)] following laparoscopic half-proximal gastrectomy (LPG), including laparoscopic total gastrectomy (L-TG; *n* = 30) as a control group, at 1 year after surgery.

**Methods:**

Clinical investigations were performed in each patient, and functional evaluations, involving the swallowing of an alimentary liquid containing acetaminophen (AAP), followed by measurements of the concentrations of AAP and hormones in the sitting (*n* = 5) and in the supine positions (*n* = 5), were carried out in each group.

**Results:**

The post-/preoperative body weight ratios were significantly higher in the L-DT and L-JIP groups than in the L-TG group. The AAP levels were significantly lower in the LPG group than in the LTG group. The AAP, insulin, and gastrin levels in the L-JIP group were markedly increased in the sitting position compared with the supine position, while those in the L-DT and L-TG groups were stable in both positions.

**Conclusions:**

L-JIP and L-DT are procedures that maintain gradual intestinal absorption and help improve the quality of life. Intestinal absorption and hormonal secretion were relatively unaffected by the posture of the meal intake after L-DT, so L-DT might be the procedure providing the most stable results.

## Introduction

The gastric cancer rate in Japan has consistently decreased over time in all age groups. However, despite this decreasing trend, the number of incident cases has been increasing due to aging of the population, and the number of deaths has remained unchanged [1]. Furthermore, the incidence rate of adenocarcinoma of the esophagus and cardia is increasing faster than that of any other malignancy [2].

Since patients are expected to survive for longer after surgery, there has been increasing demand for less invasive and safer operative procedures that are associated with an improved postoperative quality of life (QOL) [3]. For early primary gastric cancer located in the upper third of the stomach, we perform proximal gastrectomy (PG). Various methods of open or laparoscopic resection with reconstruction have been devised over time [4–6]. Standard PG for early cancer, as defined by the Japanese gastric cancer treatment guidelines [7], requires resection of less than half of the stomach. The criteria for PG in our institute were as follows: (1) a primary tumor located in the upper third of the stomach, (2) cancerous invasion not extending beyond the submucosal layer, and (3) no macroscopic evidence of lymph node metastasis at the time of surgery [8, 9]. Recently, the combination of laparoscopic gastrectomy and reconstruction has been adopted as a potentially less invasive surgical approach [10, 11]. We have been performing laparoscopic PG (LPG) for early gastric cancer, with reconstruction by the double-tract (DT) method. However, when we performed open PG, the jejunal interposition (JIP) method was adopted and contributed to a better QOL for patients, especially with respect to reduction of postoperative body weight loss, than after JIP following total gastrectomy and subtotal PG [12]. Therefore, we devised a method to change to laparoscopic JIP (L-JIP) from laparoscopic DT (L-DT) by crimping the jejunum at the anal side of the jejunogastrostomy with a knifeless linear stapler.

In the present study, the functional outcomes were prospectively compared between L-DT and L-JIP reconstruction following laparoscopic half-PG for gastric cancer, including laparoscopic total gastrectomy with Roux-en-Y reconstruction (L-TG) in the same time period as a control group. Which reconstruction resulted in a better QOL and postoperative function following proximal gastrectomy was examined.

## Patients and methods

### Study design

This study evaluated 30 patients who underwent laparoscopic PG for cancer between April 2012 and June 2016 at our institution. Resection and reconstruction were prospectively performed using L-DT and L-JIP alternately. This was accompanied by dissection of the perigastric lymph nodes up to D1+ (dissection of lymph node stations 7, 8a, 9, and 11p, in addition to the perigastric nodes) [13]. The hepatic and pyloric branches of the vagus nerve were routinely preserved, but preservation of the celiac branch was not considered. At the initial point of this study, the control group had not been determined because of the difference in indications between LPG and L-TG. It became clear that the postoperative QOL was similar between L-DT and L-JIP, and some researchers have reported that there were few significant differences in the postoperative QOL between total and proximal gastrectomy [14, 15].

Although the data were retrospective, we decided to compare LPG with L-TG as the control group. Thirty patients who underwent L-TG in the same time period were selected. The clinicopathological findings of the gastric resections were recorded according to the Japanese Classification of Gastric Carcinoma, 3rd English edition [16].

This study was approved by the Human Ethics Review Committees of Osaka Medical College and Tokai University School of Medicine (Institutional Review Board numbers 371 and 14R043, respectively). Written, informed consent was obtained from each enrolled patient before study entry in accordance with the Declaration of Helsinki.

### Outcome measures

The primary outcome measure was the postoperative digestive function as evaluated by the postoperative/preoperative body weight ratio (BWR) and the postoperative/preoperative meal intake ratio (MIR). The MIR was approximated by the mean of the total meal intake per day compared to the preoperative intake. These data were acquired at a single time point, 12 months postoperatively, through an in-house questionnaire (Table 1). In addition, the findings of patients who underwent endoscopy postoperatively at our outpatient clinic were analyzed to investigate the incidence of esophagitis, anastomotic stenosis, and the success rate of endoscopy of the remnant stomach. Endoscopic findings of esophagitis were categorized by the Los Angeles classification [17], and esophagitis ≥ Grade A was considered clinically significant. Furthermore, to clarify the quality of the operation, the surgical parameters (operation time, blood loss) and postoperative results (postoperative hospital stay duration, postoperative complications) were investigated.

**Table 1:** Questionnaire survey about postoperative body weight, meal intake and abdominal symptoms. Reprented from Nomura et al. [33]

1. Please describe your body weight at present —kg 2. Please put a circle around the number below that fits your present postoperative total meal intake per day compared to your preoperative total meal intake (1) 20% (2) 40% (3) 60% (4) 80% (5) 100% (6) Other—% 3. Please put a circle around the number below that best describes your abdominal symptoms often occurring after meals, especially at present (1) Borborygmi (2) Abdominal pain (3) Diarrhea (4) Nausea or vomiting (5) Abdominal sensation of feeling full (6) Abdominal discomfort (7) Heartburn or reflux (8) No symptoms	

### Functional evaluations

In addition, functional evaluations were performed for patients who were undergoing regular follow-up at our hospital and agreed to be involved in the study. The course of intestinal absorption and gastric non-absorbable stasis were investigated with acetaminophen (AAP) in 10 L-DT patients, 10 L-JIP patients, and 10 LTG patients, excluding patients with impaired glucose tolerance. AAP is not absorbed in the stomach but is absorbed in the duodenum or jejunum, through which it enters the blood stream [18]. Patients swallowed an alimentary liquid (200 mL of Ensure liquid mixed^®^; Meiji, Tokyo, Japan) containing 1.5 g of AAP, and the concentration of AAP in the blood was measured every 15 min for 60 min [6, 12]. At the same time, the blood concentrations of sugar, insulin, and gastrin were measured. The measurements were carried out in the sitting (*n* = 5) and in supine positions (*n* = 5) in ten patients in each group over 60 min.

### Surgical procedures

For proximal half-gastrectomy, the resection line was, in principle, at 10 cm of the lesser curvature and 15 cm of the greater curvature measured from the pyloric ring. The tumor was confirmed to be located in the upper third of the stomach preoperatively and intraoperatively. This was often ascertained through a preoperative upper gastrointestinal series or endoscopic submucosal tattooing with 0.1 mL of India ink. Two types of reconstructions following PG were performed alternately: laparoscopic proximal half-gastrectomy followed by double-tract reconstruction with a 6-cm jejunogastrostomy (L-DT) and laparoscopic proximal half-gastrectomy followed by jejunal interposition reconstruction by crimping the jejunum at the anal side of the jejunogastrostomy in L-DT with a knifeless linear stapler (L-JIP).

L-DT was performed by interposing a 15-cm segment of jejunum between the esophagus and residual stomach. In brief, the anvil head of the circular stapler (PCEEA™; Covidien, Mansfield, MA, USA) was inserted into the esophageal stump. The jejunum was divided 20 cm distal to the ligament of Treitz. A side-to-side jejunojejunostomy was created by anastomosis between the divided oral jejunum and 30 cm of anal jejunum from the oral jejunal stump. An entry hole for the circular stapler was made halfway (15 cm) along the anal jejunal stump, and the circular stapler was used to achieve esophagojejunostomy intracorporeally. After connecting the anvil head of the stapler and the circular stapler, an end-to-side esophagojejunostomy was fashioned. To clearly observe the anastomotic site without being disturbed by the circular stapler inserted through an umbilical port wound, it was thought better to insert the circular stapler through the entry hole that had been made into the jejunogastrostomy.

After removing the circular stapler, the anastomosis between the entry hole and the oral edge of the remnant stomach was made by hand sewing through an umbilical wound. The length of the jejunogastrostomy was 6 cm. For L-JIP, the jejunum at the anal side of the jejunogastrostomy was then crimped with a knifeless linear stapler (ENDOPATH^®^ ETS 45-mm No Knife; Ethicon Endo-Surgery, Cincinnati, OH, USA).

**Fig. 1:**
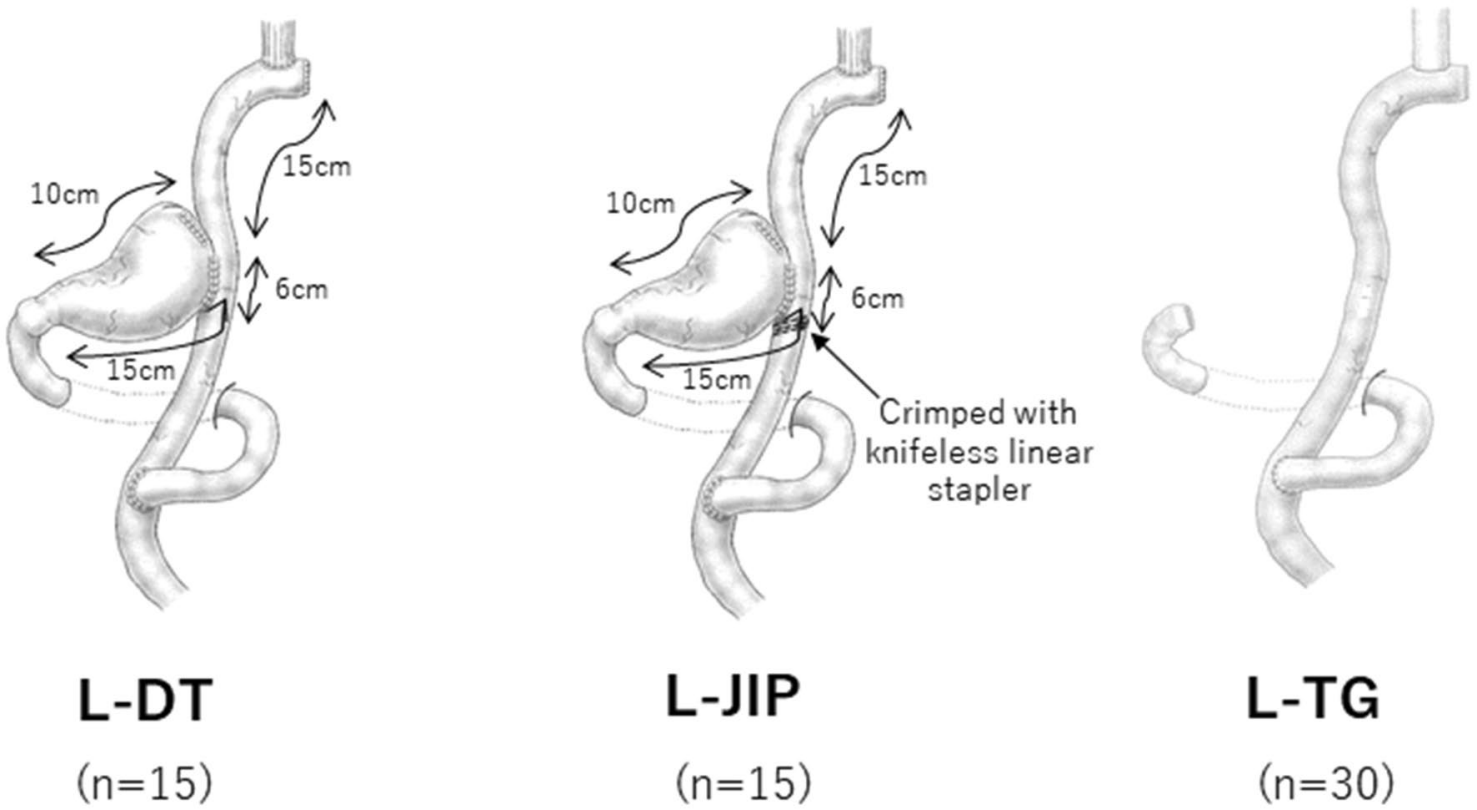
Schematic illustrations of the surgical procedures. L-DT: laparoscopic double-tract reconstruction following proximal half-gastrectomy. *L-JIP* laparoscopic jejunal interposition reconstruction following proximal half-gastrectomy, *L-TG* laparoscopic Roux-en-Y reconstruction following total gastrectomy. Reprented from Nomura et al. [33]

The criteria for L-TG in our institute were as follows: (1) widespread early gastric cancer beyond the upper third of the stomach, (2) multiple early gastric cancers located in the upper third and other regions, and (3) no macroscopic evidence of lymph node metastasis. L-TG was performed by interposing a 35-cm segment of jejunum between the esophagus and the jejunojejunostomy using the circular stapler, as well as in L-DT and L-JIP. This was accompanied by dissection of the perigastric lymph nodes up to D2 without splenectomy. The hepatic and pyloric branches of the vagus nerve were not preserved. These procedures are illustrated in Fig. 1.

### Statistical analyses

Statistical analyses were performed using the JMP^®^ software program, ver. 13 (SAS Institute Inc., Cary, NC, USA). Student’s *t* test and the *χ*^2^ test were used for comparisons. A *p* value of less than 0.05 was considered significant.

## Results

### Patients’ clinical characteristics

Of the 30 patients who underwent LPG, 15 underwent L-DT, and 15 underwent L-JIP, while another 30 underwent L-TG. All patients completed the digestive function questionnaires. Patient demographics, stratified according to the surgical procedure, are presented in Table 2; there were significant differences in the number of cases over stage IIA between the L-TG and LPG groups (*p* = 0.020), especially in the L-DT group (*p* = 0.018), even though L-TG had been performed according to our indication in the same time period. Adjuvant chemotherapy with S-1 was given to one case in the L-JIP group and six cases in the L-TG group. However, follow-up showed that there was no evidence of recurrence at 1 year after surgery in any patients.

**Table 2:** Characteristics of patients by type of reconstruction

	L-DT	L-JIP	LPG	L-TG
Sex (male:female)	13:2 (*n* = 15)	11:4 (*n* = 15)	24:6 (*n* = 30)	21:9 (*n* = 30)
Age (years old)	65.7 ± 10.6	69.3 ± 6.0	67.5 ± 8.7	68.5 ± 8.3
Stage (cases)				
IA	11	11	22	17
IB	4	2	6	4
IIA	0	2	2	3
IIB	0	0	0	5
IIIA	0	0	0	1

There were significant differences in the number of cases over stage IIA between the L-TG and LPG groups (*p* = 0.020). Adjuvant chemotherapies with S-1 were given to one in the L-JIP group and six cases in the L-TG group

### Surgical parameters and postoperative results

Surgical parameters and postoperative results are listed in Table 3. There were no significant differences among the three groups in the surgical parameters. However, the postoperative hospital stay was significantly longer in the L-TG group than in the LPG group. This might have been caused by postoperative complications for which the recovery time was long, such as anastomotic leakage and pancreatic fistula.

**Table 3:** Surgical parameters and postoperative results

	L-DT (*n* = 15)	L-JIP (*n* = 15)	LPG (*n* = 30)	L-TG (*n* = 30)
Operation duration (min)	352.5 ± 67.3	322.5 ± 24.2	337.5 ± 52.0	341.9 ± 51.3
Blood loss (ml)	90.5 ± 105.5	46.8 ± 69.8	68.7 ± 90.7	86.1 ± 90.8
Postoperative hospital stay duration (day)	14.2 ± 4.4	14.7 ± 5.1	14.4 ± 4.6^a^	19.3 ± 11.0^a^
Postoperative complications (cases)				
Anastomotic leakage	0	0	0	2
Pancreatic fistula	0	1	1	4
Hemorrhaging	1	0	1	1
Anastomotic stenosis	1	1	2	1
Cholecystitis	1	0	1	0

^a^There was a significant difference in the duration of postoperative hospital stay between the LPG and L-TG groups

The items are not in the correct position in Table 3

### Clinical outcomes at 12 months

**Fig. 2:**
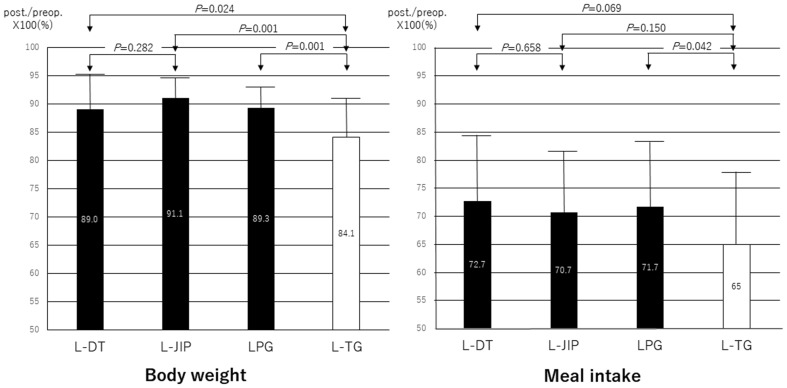
Postoperative/preoperative body weight and meal intake ratios. The postoperative/preoperative body weight ratios were significantly higher in the L-JIP and L-DT groups than in the L-TG group. While a comparison of the postoperative/preoperative meal intake ratio showed no significant difference among the three groups, it tended to be higher in the L-DT group than in the L-TG group (*p* = 0.069)

While a comparison of the MIR showed no significant difference among the three groups, it tended to be higher in the L-DT group than in the L-TG group (*p* = 0.069). The BWR was significantly higher in the L-JIP and L-DT groups than in the L-TG group (Fig. 2). Furthermore, when seven cases who received S-1 chemotherapy were excluded, the BWR was significantly higher in the L-JIP (91.2 ± 3.7) and L-DT (89.0 ± 6.3) groups than in the L-TG (83.8 ± 7.4) group. However, the MIR showed no significant difference between the L-DT (72.7 ± 13.4) group and the L-TG (65.4 ± 14.1; *p* = 0.120) group or between the LPG (71.4 ± 12.2) group and the L-TG group (*p* = 0.105). With respect to postprandial symptoms, an abdominal sensation of fullness was frequent in all groups: 26.7% (4/15) in L-DT, 40.0% (6/15) in L-JIP, and 30.0% (9/30) in L-TG. Abdominal discomfort (20.0%, 3/15) in L-DT, borborygmi and abdominal pain (20.0%, 3/15) in L-JIP, and heartburn (16.7%, 5/30) in L-TG were the second most frequent symptoms. Abdominal symptoms of any sort were present in 53.3% (8/15) of patients in the L-DT and L-JIP groups and 60.0% (18/30) of those in the L-TG group. However, there were no significant differences among the three groups.

### Findings on endoscopic examinations

The incidence of reflux esophagitis on endoscopic examinations in the L-DT, L-JIP, and L-TG groups was 6.7% (1/15), 6.7% (1/15), and 3.3% (1/30), respectively. The incidence of residual food retention in remnant stomach in the L-DT and L-JIP groups was 13.3% (2/15) and 26.7% (4/15), respectively. Stenosis of the esophagojejunostomy was observed in two patients each (13.3%, 2/15) in the L-DT and L-JIP groups and in one patient (3.3%, 1/30) in the L-TG group, but the stenoses were improved by a single-balloon dilatation. The endoscope was able to reach the remnant distal stomach in all PG patients. However, there were no patients who had a severe esophageal hiatal hernia preoperatively.

### Functional outcomes at 12 months

**Fig. 3:**
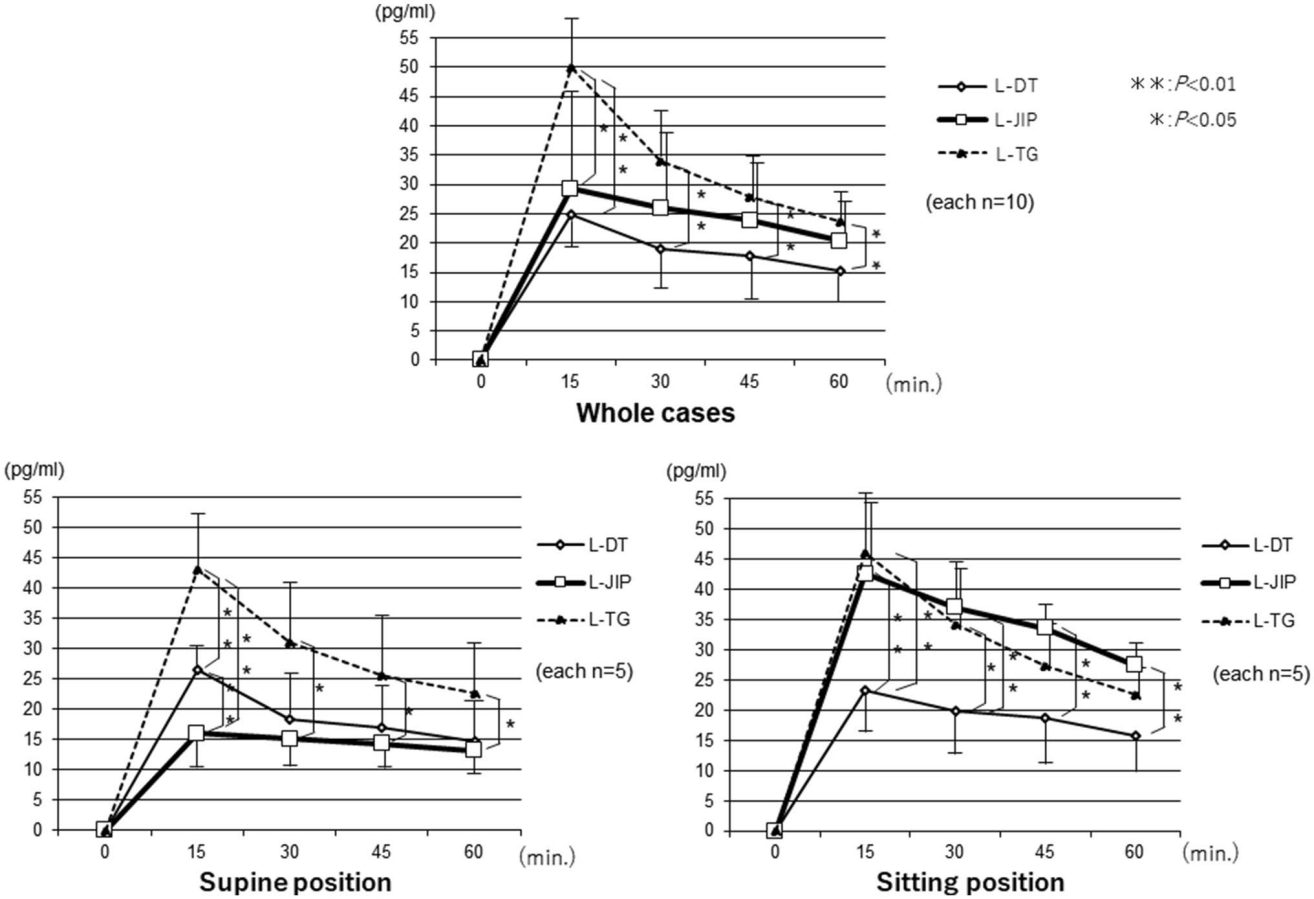
Changes in the plasma acetaminophen concentrations. The changes in the plasma AAP concentration were significantly milder in the LPG group than in the L-TG group. The AAP curve in the sitting position in the L-JIP group was shifted upward from that in the supine position. The number of cases in each group was ten in total, with five each in the supine and sitting positions. **p < 0.01, *p < 0.05

**Fig. 4:**
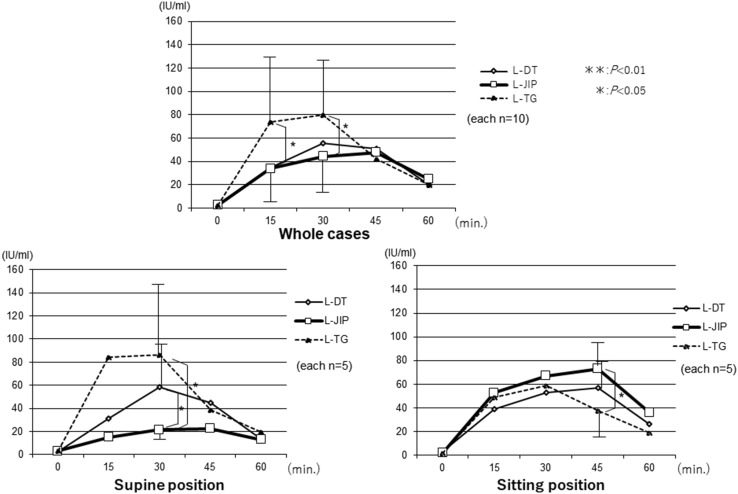
Changes in the postprandial insulin levels. The curves of the plasma insulin levels resemble the curves of the plasma AAP concentrations. **p < 0.01, *p < 0.05

**Fig. 5:**
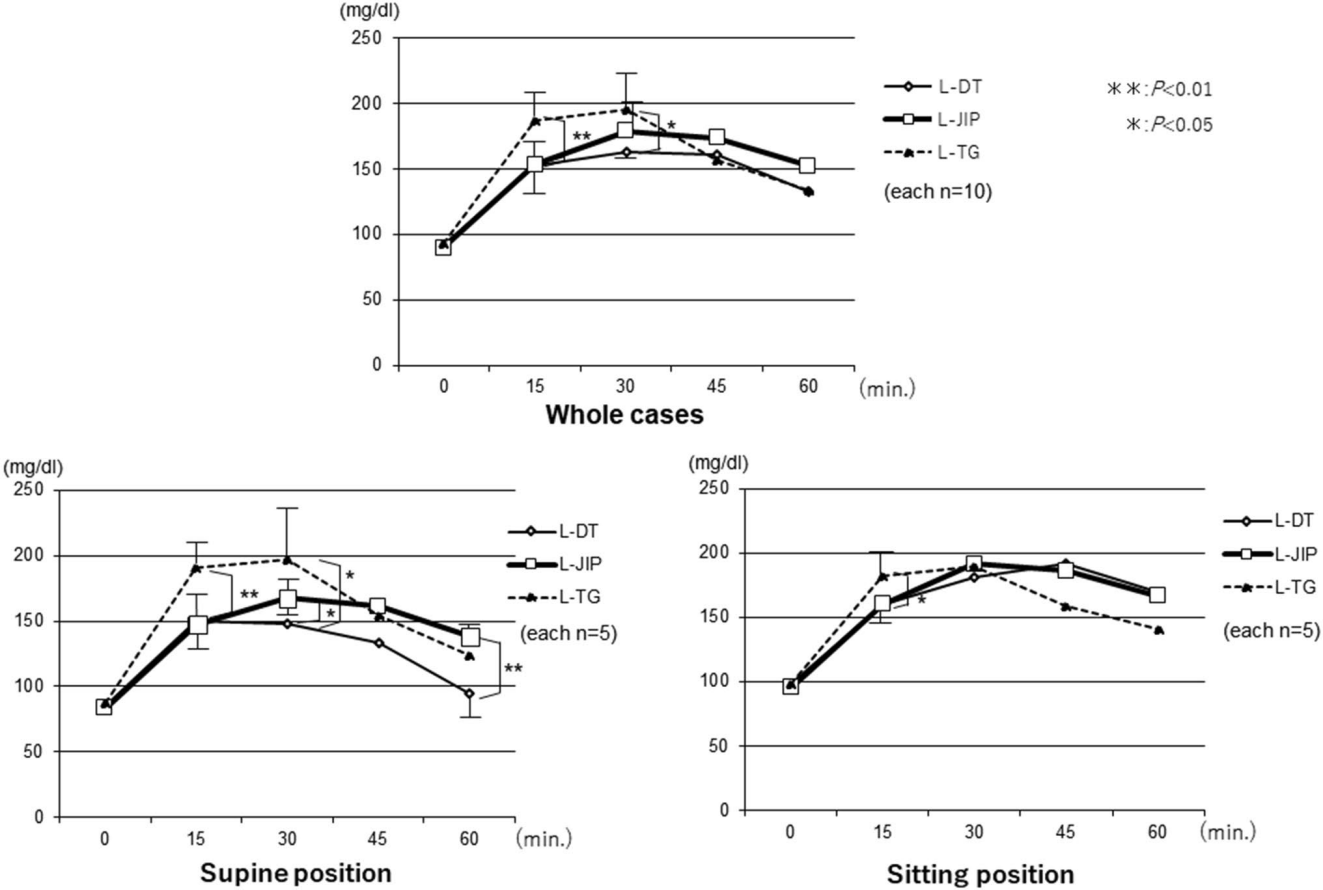
Changes in the postprandial blood sugar levels. In the sitting position, the blood sugar levels showed a more gradual increase in the L-DT and L-JIP groups than in the L-TG group, but in the supine position, those in the L-DT group showed a decrease. **p < 0.01, *p < 0.05

**Fig. 6:**
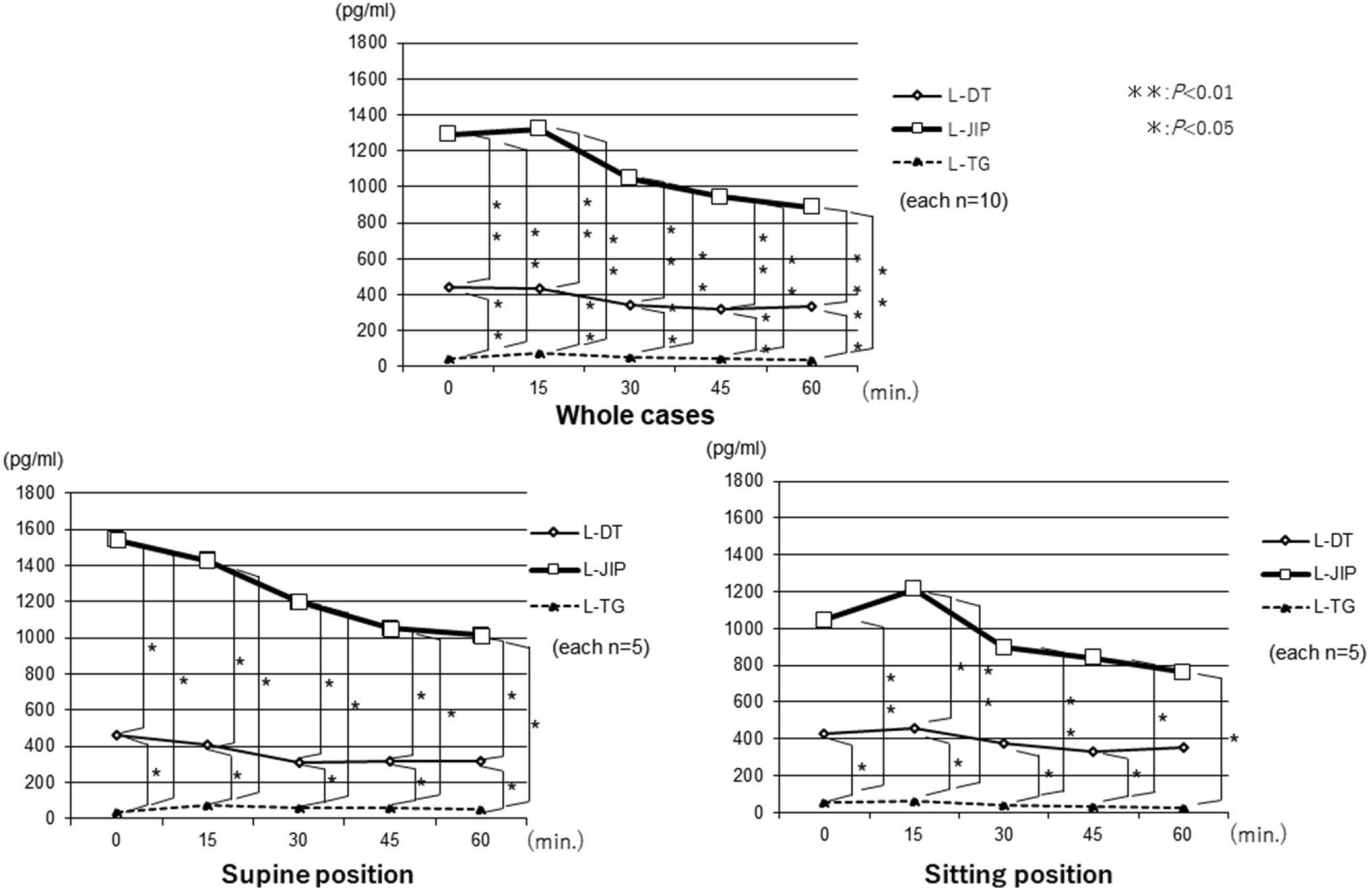
Changes in the postprandial gastrin levels. The plasma gastrin levels were the highest in the L-JIP group, about one-third the level of the L-JIP group in the L-DT group, and the lowest in the L-TG group. **p < 0.01, *p < 0.05

The changes in the AAP levels were significantly lower in the LPG groups than in the L-TG group. Furthermore, the changes in the AAP levels in the three groups were compared between the sitting and supine positions, and the AAP levels in the L-JIP group were found to be markedly increased in the sitting position, while those in the L-DT and L-TG groups were stable in both positions (Fig. 3). The changes in the plasma insulin levels were similar to those in the AAP levels, and the insulin levels in the L-JIP group were increased to a greater degree than in the other groups from the supine position to the sitting position (Fig. 4). In contrast, the changes in the blood sugar levels in the L-DT group were significantly lower than those in the other groups in the supine position (Fig. 5). However, the changes in the plasma gastrin levels were almost the same in both positions (Fig. 6).

## Discussion

The incidence rate of lower esophageal adenocarcinoma was significantly reduced by *Helicobacter pylori* infection in Japan. If the *H. pylori* infection rate were to decrease, the incidence rate of cancer in the esophagogastric junction or upper part of the stomach might increase in the future [2]. Therefore, PG is now attracting attention.

The QOL after PG has been intensively evaluated over time. Takiguchi et al. [19] reported that PG was better than total gastrectomy in terms of weight loss, need for additional meals, diarrhea, and dumping symptoms in a multi-institutional study. Especially in esophagogastrostomy after PG, Inada et al. [20] reported that diarrhea scores and the need for additional meals were lower in the group with more than three-quarters of a remnant stomach than in those with a remnant stomach two-thirds the preoperative size. In the present study, the postoperative body weight and meal intake were significantly higher in the LPG group than in the L-TG group.

Three reconstructive procedures need to be considered: jejunal interposition reconstruction, double-tract reconstruction, and esophagogastrostomy. The first two methods involve reconstruction with 8–15 cm of interposed jejunum between the esophagus and the remnant stomach to prevent reflux esophagitis and to observe the remnant stomach for follow-up of neoplastic tumor [21–23]. The third method involves reconstruction by fundoplication, wrapping the remnant stomach around the circumference of the esophagus [24], by the double-flap technique and embedding the lower edge of the esophagus in the gastric submucosal layer [25]. The QOL for each of these procedures has recently been evaluated.

In the present study, the functional outcomes were prospectively compared between two types of reconstruction following LPG, including L-TG as a control group. The BWR was significantly higher in the L-DT and L-JIP groups than in the L-TG group, whether patients who received S-1 chemotherapy were included or excluded. Furthermore, the MIR tended to be higher in the L-DT group than in the L-TG group. However, when S-1 chemotherapy cases were excluded, this tendency disappeared. While we must take into account the effect of adjuvant chemotherapy on the QOL [26], the BWR was not affected by S-1. This seems to be due to the postoperative stabilization at 1 year after surgery [27] and the completion of chemotherapy.

However, the causes of these differences have not been reported, so the observation of the biological reactions after nutritional loading is thought to be useful. Intestinal absorption using the AAP method and the kinetics of hormonal secretion were evaluated. Furthermore, by evaluating the changes in the sitting and supine positions, observation of the intracorporeal changes with mainly peristaltic intestinal motion in the supine position as well as with both peristaltic and gravitational intestinal motion in the sitting position, which is physiological at the time of the meal intake, was believed to be possible. The changes in the plasma AAP concentrations were found to be significantly milder in the LPG group than in the L-TG group. Furthermore, concerning the changes in each position, there were relatively few changes in the AAP curve in the L-TG and L-DT groups. In contrast, however, the AAP curve in the L-JIP group was shifted upward from the supine position to the sitting position. These differences might have been caused by the gravitational intestinal motion in the sitting position.

The plasma gastrin levels were highest in the L-JIP group, about one-third the level of the L-JIP group in the L-DT group, and lowest in the L-TG group. However, changes in the curves of plasma gastrin levels with posture were almost indiscernible. Whether or not hypergastrinemia has a good effect remains unclear at present. Gastrin also acts as a potent cell-growth factor that has been implicated in a variety of normal and abnormal biological processes, including maintenance of the gastric mucosa, proliferation of enterochromaffin-like cells, and neoplastic transformation [28]. Tosetti et al. [29] reported that delayed gastric emptying is related to increasing serum gastrin levels, according to a survey of fundic atrophic gastritis. Although it was in the postoperative state, remnant gastric emptying was reduced by the effects of hypergastrinemia, thus the liquid diet might have been promptly emptied solely by the effects of gravity.

In addition, the curves of the plasma insulin levels resembled the curves of plasma AAP concentrations. In the sitting position, the blood sugar levels showed a more gradual increase in the L-DT and L-JIP groups than in the L-TG group; however, in the supine position, the levels in the L-DT group showed a decrease. These results are similar to those seen with bariatric surgery for morbid obesity, which causes insulin secretion or sensitivity to improve. Hormones, such as incretins secreted from the small intestine, may accelerate insulin secretion and suppress blood sugar changes [30, 31]. Based on these results, the kinetics of intestinal absorption and hormonal secretion were deemed stable in L-DT, suggesting that the procedure may be superior to L-JIP as function-preserving gastrectomy. The details of this mechanism are unclear, but if excessively large meals enter the remnant stomach, they may overflow into the efferent jejunal route. Such mechanisms may contribute to the stability of the kinetics and increased meal intake. Nevertheless, the BWR showed no significant differences between the two groups. This might be caused by the nutritional advantages obtained due to meal passage through the duodenum [32].

Several limitations associated with the present study warrant mention. The sample size was relatively small, and the AAP method is primarily an indirect investigation of the gastric emptying function and uses only a liquid meal, even though solid meals are usually taken. How a solid meal is absorbed and metabolized remains unknown, and the mechanism needs to be elucidated in a future study. Furthermore, it was not possible to examine the relationships between these hormones and gastroduodenal digestive juices, especially gastric, pancreatic, and bile juices. However, it was thought that the ease of intestinal absorption and the status of hormonal secretion of each operative method could be evaluated and identified.

In conclusion, L-JIP and L-DT are procedures that nearly completely maintain the preoperative gradual intestinal absorption, and they achieve a better postoperative QOL as function-preserving procedures than L-TG. Furthermore, the intestinal absorption and hormonal secretion in the L-DT group were largely unaffected by the posture of the meal intake, suggesting that L-DT might be the procedure providing the most stable results. Further randomized clinical trials comparing L-DT and L-JIP will be needed to verify various functions in detail, including investigations of hormones, such as incretins, and gastroduodenal digestive juices.

## Abbreviations

QOL: Quality of life
PG: Proximal gastrectomy
LPG: Laparoscopic proximal gastrectomy
DT: Double-tract reconstruction
JIP: Jejunal interposition reconstruction
L-DT: Laparoscopic double-tract reconstruction
L-JIP: Laparoscopic jejunal interposition reconstruction
L-TG: Laparoscopic total gastrectomy
BWR: Postoperative/preoperative body weight ratio
MIR: Postoperative/preoperative meal intake ratio
AAP: Acetaminophen
D2: D2 lymphadenectomy
*H. pylori*: *Helicobacter pylori*
cN0: No regional lymph node metastasis macroscopically
cT1: Tumor confined to the mucosa or submucosa macroscopically
